# Experiment Analysis on Crack Resistance in Negative Moment Zone of Steel–Concrete Composite Continuous Girder Improved by Interfacial Slip

**DOI:** 10.3390/ma15238319

**Published:** 2022-11-23

**Authors:** Wenqing Wu, Jinxi Dai, Liang Chen, Dan Liu, Xiaoyi Zhou

**Affiliations:** 1 School of Transportation, Southeast University, Nanjing 211189, China; 2 China Railway Eryuan Engineering Group Co., Ltd., Chongqing Survey and Design Institute Co., Ltd., Chongqing 400023, China; 3 Chengxian College, Southeast University, Nanjing 210088, China

**Keywords:** bridge engineering, crack resistance, experimental beam, steel–concrete composite structures, negative bending moment zone, interface slip

## Abstract

Due to the strong interface effect of continuous steel–concrete composite beams with conventional shear connectors, the efficiency of applying pre-stress in the negative moment zone is greatly reduced, which leads to a difficulty of anti-cracking design in the negative moment zone of pre-stressed steel–concrete composite box girder. In order to study the feasibility and the working mechanism of improving the crack resistance of continuous steel–concrete composite bridges by releasing the interfacial slip effect within the negative bending moment region, two groups of model tests were carried out in the paper. Two steel–concrete composite beams were used for model test, one of them using the conventional stud shear connectors, another one using the new shear connectors, named uplift-restricted and slip-permitted shear connectors. The results show that, compared with the composite beam with conventional shear studs, the composite beams with uplift-restricted and slip-permitted shear connectors have a higher pre-stress application efficiency, and the new connector could release the interface slip, which can make the tensile stress distribution in concrete slab more uniform within the negative moment zone, thus increasing the cracking load of concrete slab and reducing the subsequent crack width effectively. This study is helpful to understand the relationship between the interface slip and the anti-crack characteristics in negative moment zones, and a new anti-crack design method is proposed for the design of continuous composite girder.

## 1. Introduction

The steel–concrete continuous composite girders have many obvious structural advantages over the simply supported composite beams, but they are confronted with the design problem of anti-cracking of concrete bridge deck in negative moment zones. Because the concrete bridge deck is under the tensile state, it is easy to crack. Furthermore, the rainwater may infiltrate into concrete to corrode the shear connectors; thus, the durability of the composite structure is reduced.

Previous research showed that there are two kinds of crack-resisting design methods for steel–concrete composite continuous beams, especially focusing on the hogging bending moment zone. One is to allow for the cracking of concrete bridge decks, but the crack width could be limited, based on the numerical simulation to the crack development process [1,2], such as taking a design method of reinforcement encryption in the hogging moment zone. With the development of material technology, many scholars have proposed the use of concrete with better tensile performance than normal concrete. Fan et al. used an engineered cementitious composite to improve the cracking load [3]. Qi et al. and Zhang et al. combined ultra-high-performance concrete with steel beams, significantly improving the bearing capacity and crack resistance of composite structures [4,5]. The other is to take structural measures to eliminate the tensile stress of concrete bridge decks and reduce the possibility of concrete cracking. Some common methods include jacking up/down construction at the intermediate support, adjusting the longitudinal concrete pouring sequence of bridge decks, arranging prestressed reinforcement in negative moment zone, etc. Among the above methods, the structural design measures used commonly are mainly to apply prestress in the bridge deck within the negative moment zone, which can not only enhance the crack resistance of the structures [6], but also give the structure a longer fatigue life [7]. However, owing to the existence of shear connectors between steel and concrete, a considerable part of the prestress will be transmitted to the steel portion, resulting in an insufficient effective prestress in the concrete bridge decks and some difficulties in effectively enhancing the crack resistance of the structures. Therefore, it is urgent to put forward a new mechanics concept for a better design of steel–concrete composite continuous girders.

At present, there are two conventional types of shear connectors, named the stud connector and perfobond strip connector, that are widely used in simply supported steel–concrete composite girders [8]. Because the interfacial slip is restricted, the shear force could be transferred; hence, these two groups of connectors were termed as slip-restricted connectors [9,10,11,12,13].

There are many literatures showing that the interfacial slip is not conducive to the mechanical performance of the simply supported composite structure, which will lead to the reduction of the structure stiffness, the increase of deformation, and the reduction of the structure bearing capacity [14,15,16,17]. Therefore, the relative interfacial sliding between steel and concrete needs to be avoided in the simply supported structural design, which is helpful for realizing the compatible deformation of steel and concrete and improving the flexural stiffness and flexural bearing capacity of the structures. The above viewpoint is also applicable to the flexural design of steel–concrete composite continuous beams in the positive moment zone. In order to reflect the adverse effect of interface slip on the structural performance, many researchers carried out numerical simulation analyses on the interface slip behavior of composite girder [18,19,20,21] and proposed a calculation method of a composite girder, considering the interface slip effect [22,23,24], with a view towards considering the adverse impact of the interface slip on the structural stiffness, stress, and bearing capacity. Therefore, the mainstream academic view holds that interface slip has an obvious negative effect on the stress state of simply supported composite girder. The same point of view also applies to the flexural characteristics in the positive moment zone of the composite continuous beam. Therefore, how to understand the different effects of interface slip between the structural mechanical properties on the positive moment zone and on the negative moment zone has become a huge obstacle to effectively deal with the anti-crack design of bridge decks in the negative moment zone. This paper will put forward a reasonable understanding, as well as analysis results, based on the model test data.

It is well-known that the combination of the steel portion at the bottom layer and concrete slab at the upper layer can be said to perfectly meet the structural stress requirements, giving full play to the respective characteristics of the two types of materials in the positive bending moment region of continuous composite girders. However, the concrete slab in the region of negative bending moment is under tensile mechanical states, and a high level of anti-crack resistance becomes a key issue for the design of a continuous composite girder. Therefore, the focus of structural design in the negative moment zone is obviously different from that in the positive moment zone, and there should be different design concepts, so as to effectively solve the problem that the concrete slab in the negative moment zone is easy to crack.

Based on the above preliminary understanding to the combination of steel and concrete, the authors of the paper consider that, since the shear action of the shear connectors leads to the transferring of prestress from the concrete bridge deck to the steel portion, then the available effective way to improve the efficiency of prestressing is to produce enough interface slip, rather than limit it. The method mentioned above may be an effective method to enhance the crack resistance of concrete slab in the region of the negative bending moment.

**Figure 1:**
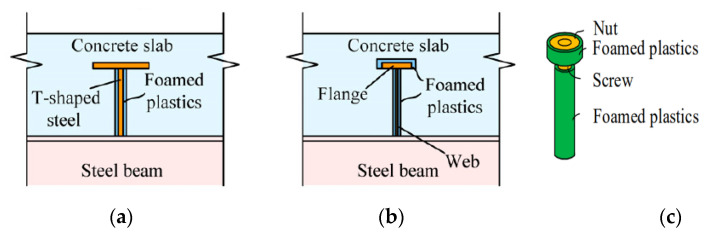
Common shear connectors used in steel–concrete composite bridge. (**a**) FS connector, (**b**) URSP-T connector, (**c**) URSP-S connector.

The effective way to reduce tensile stress in concrete is to release the shearing force between the steel and concrete, instead of restricting it. However, there is little literature on enhancing the crack resistance of bridge deck in the region of negative bending moment by reducing interfacial shear action. A flexible shear member for a composite girder bridge was proposed by Abe and Hosaka [25] (Figure 1a), herein T-section steel wrapped with foamed plastics was welded to the top flange of the steel beam, providing an uplift-restricted function, but not an anti-shearing function. Because the stiffness of foamed plastics is much lower than that of steel or concrete, the connector can permit slip between the concrete slab and steel beam and release the tensile stress inside concrete in the hogging moment region of composite beams. The shear element works by using foam to eliminate shear force, but the top surface of the connector still transmits some shear force.

In order to further eliminate the adverse effect of shear connectors mentioned above, a new novel shear connector was put forward by covering foam material not only on both sides of the shear element but also on the flange plate at the top of the shear element, so as to increase the sliding deformation between the shear element and concrete thoroughly [9,10,26]. The new shear members are named the uplift-restricted and slip-permitted T-shape connectors (URSP-T connector) (Figure 1b) and applied to the negative moment zone of a real continuous composite girder bridge. Due to the complex structure of URSP-T connector, some studies have proposed a more simplified structure, such as URSP screw-shaped connectors, which are more adaptable to the requirements of rapid construction than URSP T-shaped connectors (Figure 1c) [27]. The application of the URSP connector was also studied to be used in steel–concrete composite frame buildings successfully, which has covered a research blank in this field [28]. The above novel structure has been applied preliminarily, but the working mechanism of a new structure still needs to be further studied to understand and improve the design method in essence.

The feasibility of the new connectors to improve the crack resistance in the region of a negative moment has been confirmed by the existing experimental studies [9,10,16,19,29,30]. However, the research about the influence path of interface slip on the crack resistance of composite girder and the related working mechanism are still insufficient, and there is still little related research literature that can be referenced, so it is necessary to carry out more experimental study in-depth. Therefore, in this paper, model tests focusing on negative moment zones were designed to, firstly, verify the effectiveness of interface slip effect on improving the crack resistance of continuous composite structures. Then, the working mechanism of the novel shear connectors was investigated, which explains the interface slip to enhance the crack resistance of the structure under two conditions, both applying prestress in the negative moment region and external negative bending moment near the mediate support of continuous composite girder.

## 2. Preparation of Experimental Scheme

### 2.1. Specimen Design

Based on the model test of two steel–concrete composite box girders with vertical webs, the stress characteristics near the middle support of continuous girder are simulated, including one ordinary experimental beam with stud connectors (named SRB, i.e., slip-restricted beam) and another experimental beam with URSP connectors (named SPB, i.e., slip-permitted beam).

**Figure 2:**
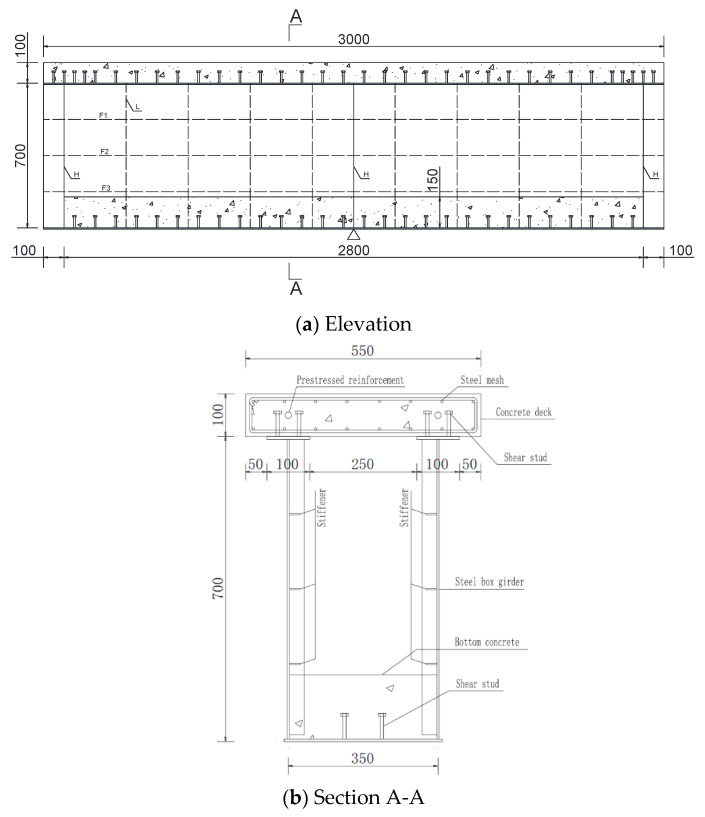
Structure diagram of the experimental beam (Unit: mm).

Specimen SRB and specimen SPB have the same dimensions and structure frames, but some differences in connectors. The shear connectors of two test specimens were different, and the detail sizes of them are shown in Figure 2. The steel portion and the upper concrete slab form a conventional composite structure, which is the main load-carrying structure, and in order to improve the local buckling stability of the bottom plate of steel portion under the action of negative bending moment, concrete was poured with a thickness of 15 cm on the bottom plate of steel portion. The above structural form is also known as double-combination structure.

The size detail of the model test beam is referred to in the relevant literature [1], and the steel portion of composite girder had a uniform cross-section with a length of 3 m, a height of 70 cm, and a width of 35 cm. The thickness of flange plate and web of steel portion is 6 mm. The diaphragms H were arranged at the intermediate support and two sections 10 cm close to the support at both ends, and rib L, F1, F2, F3 were stiffeners of steel portion with a thickness of 4 mm (Figure 2a).

Based on the structural design mentioned above, the concrete slab of composite girder is equipped with unbonded prestressed steel bars, the cross-section of concrete slab has a width of 550 mm and a height of 100 mm. The top and bottom concrete slabs are, respectively, equipped with eight ordinary steel bars along the longitudinal direction, each steel bar has a diameter of 8 mm, and with a number of ordinary steel bars along the transverse direction, each has a diameter of 6 mm. In addition to the ordinary steel bars mentioned above, the upper concrete slab is also equipped with a stirrup with a diameter of 6 mm and with a longitudinal spacing of 10 cm. The concrete slab has a reinforcement ratio of 1.462% along the longitudinal direction. In addition to ordinary reinforcement, the upper concrete slab is also equipped with prestressed reinforcement, $\phi^{s}$ 15.24 unbonded prestressed tendon.

Based on the above structure states, it can be calculated that the bending stiffness EI of the steel portion (including bottom concrete contribution) is 1.43 × 10^14^ N·mm^2^ at the intermediate support section, while the bending stiffness EI of the upper concrete slab is 1.58 × 10^12^ N·mm^2^. The flexural stiffness ratio of steel portion-to-upper concrete slab is 90.60.

**Figure 3:**
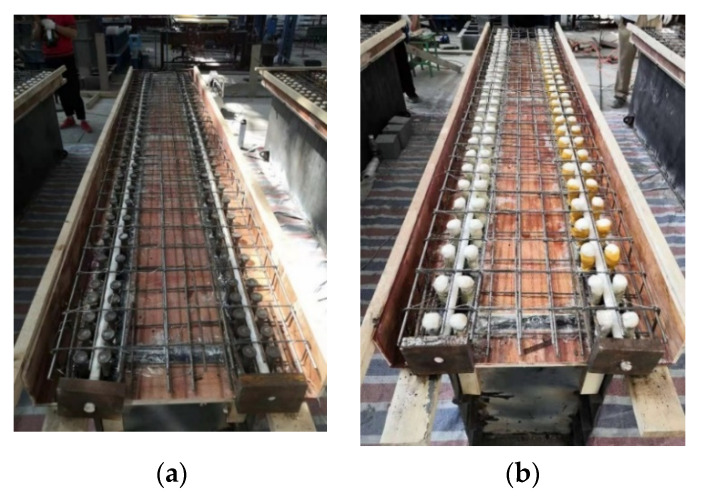
Schematic Diagram for Two Types of Shear Connectors. (**a**) conventional connector for SRB, (**b**) URSP connector for SPB.

The difference between the two experimental beams lies in the different forms of shear connectors. In the specimen SRB, the stud connectors with a diameter of 16 mm are welded on the upper flange plate of the steel portion, with a spacing of 10 cm, but encrypted at both ends, as shown in Figure 3a.

In the specimen SPB, the EVA (Ethylene-vinyl acetate) foam is wrapped around the traditional studs, which are welded on the upper flange of the steel portion to form an uplift-restricted and slip-permitted connector (named URSP connectors) [9], as shown in Figure 2b. Because the slippage in actual engineering is very small, generally not more than 2 mm, the thickness of the foam wrapped in the experiment is about 5 mm.

### 2.2. Material Performance

The material performance is listed in Table 1, Table 2 and Table 3 for test specimens.

### 2.3. Loading Conditions

**Figure 4:**
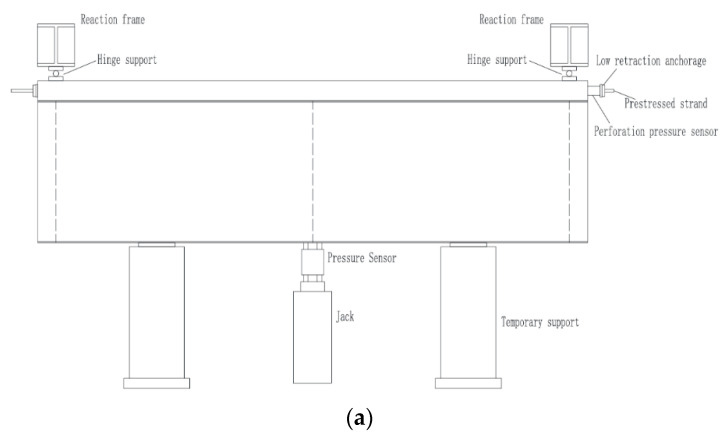

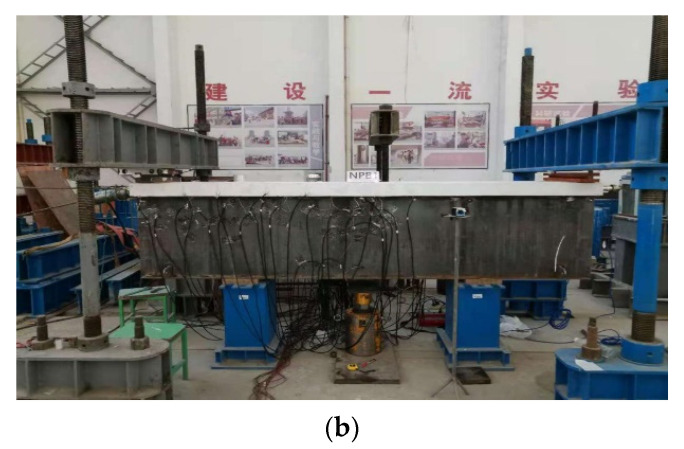
Experimental loading device. (**a**) Loading arrangement, (**b**) Field loading diagram.

The loading devices of two specimens SRB are shown in Figure 4. The loading condition was used to simulate the hogging bending moment in the intermediate support section of continuous girder.

The loading process was divided into two loading conditions, as follows.

(1) Tensioning prestress bars of the upper concrete slab

The two experimental beams were placed on the temporary supports, the prestressed reinforcements were pretensioned before loading, and the perforated pressure sensors were used to monitor the preload value. After the completion of prestress tensioning, the effective preload obtained through monitoring was 150 kN. At the same time, the structural strains of test beam and the interface slip of key sections along the longitudinal direction were all measured.

(2) Loading upward in the mid-span section of test beam

After the prestress was applied, an upward load was exerted by a jack, and the two ends of the experimental specimens were restrained by the roller support. In order to test the applicability of the test system, the specimen was preloaded to about 60 kN. Step loading was adopted during the loading processing. The loading value of each step of the jack was 20 kN, which was stopped when the loading value reached 980 kN. After 5–10 min, when the loading value was unchanged, the value of load, load deformation, the interface slip, structural strain, and tension of the prestressed steel bar was recorded for each step.

### 2.4. Layout of Measuring Points

**Figure 5:**
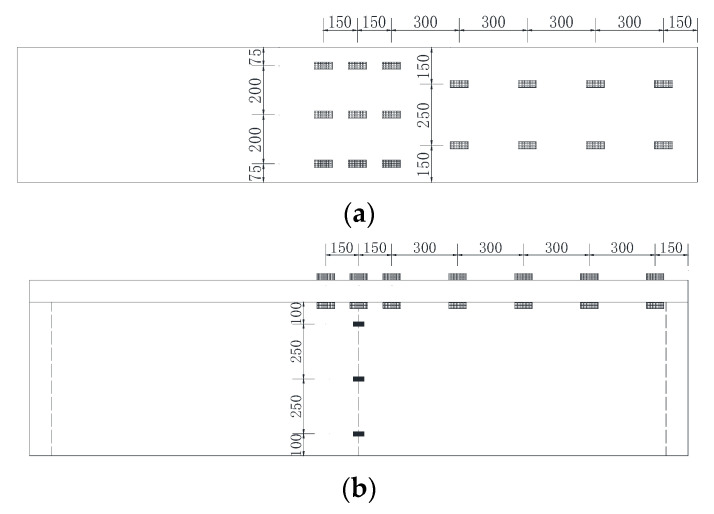

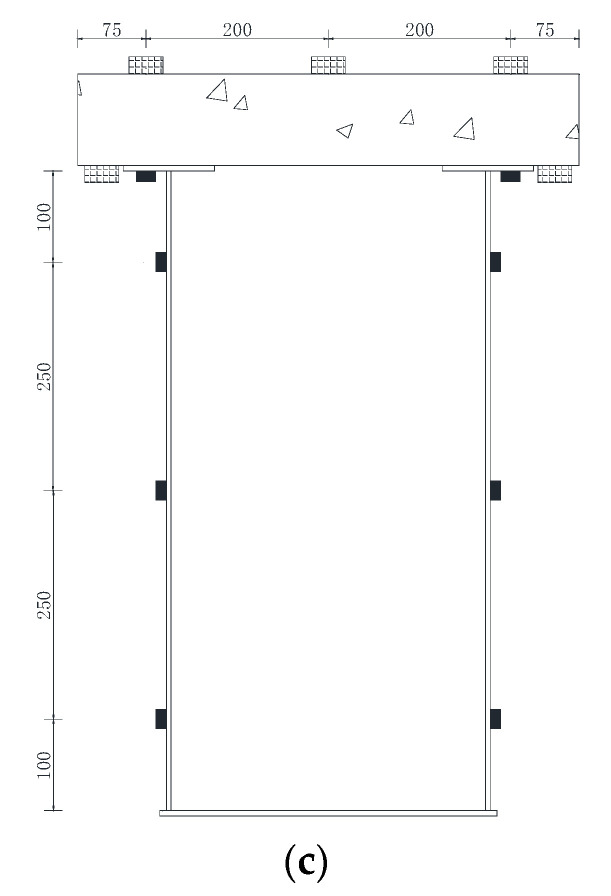
Layout of Strain Gauge on specimens SRB and SPB (unit: mm). (**a**) Top view of strain gauge on top surface of concrete slab, (**b**) Elevation view of strain gauge on concrete slab and steel box girder web, (**c**) layout of strain gauge at intermediate support section.

The arrangements of strain gauges and displacement gauges of each specimen are consistent, and the strain gauges position is shown in Figure 5.

**Figure 6:**
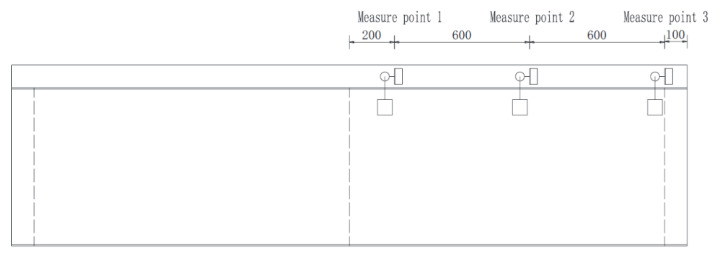
Layout of Dial Gauge (unit: mm).

**Figure 7:**
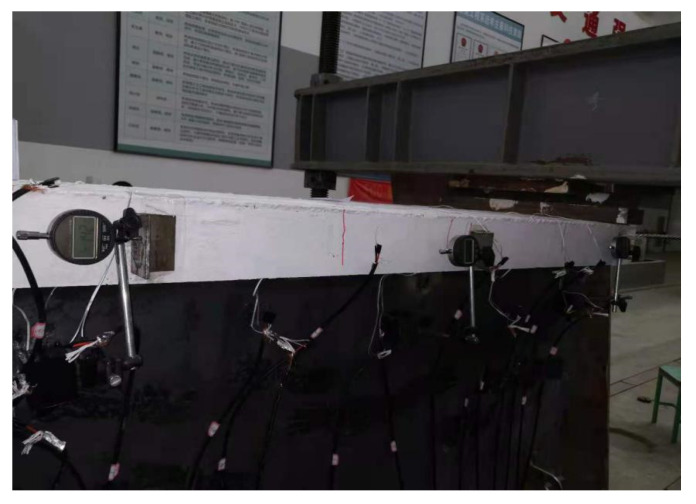
Real Fixing Method of Dial Gauge.

Three dial gauges for displacement meters were employed in three key sections to measure the relative interface slip. The specific layout and fixing method are shown in Figure 6 and Figure 7.

## 3. Analysis of Crack Resistance by Experimental Results

(1) Specimen SRB

**Figure 8:**
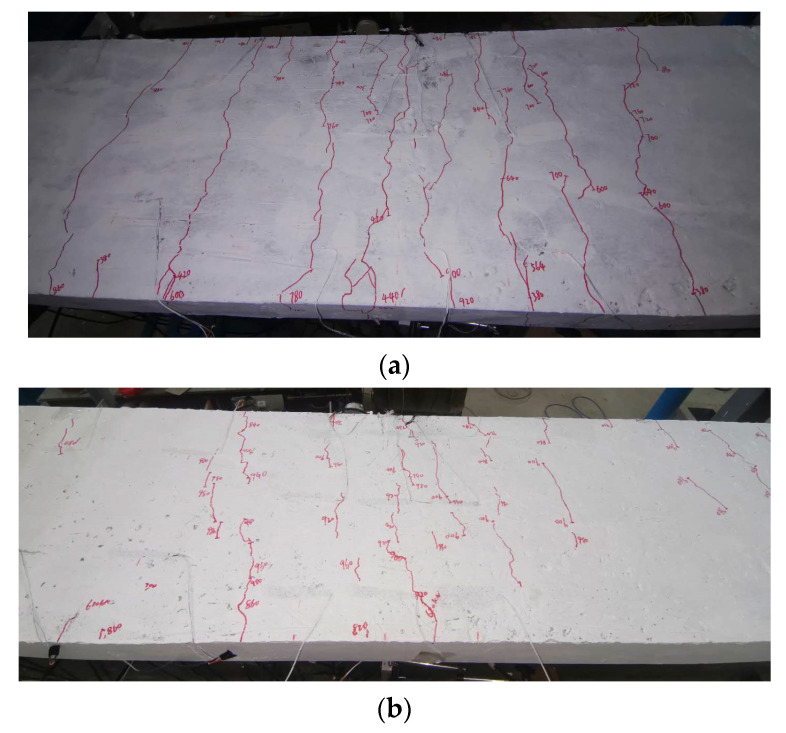
The crack distribution of the experimental beam. (**a**) Specimen SRB, (**b**) Specimen SPB.

The specimen behaved in an elastic state at the beginning of loading. When the load reached 300 kN, applied by the jack, some short cracks occurred in the upper slab near the mid-span section of the test beam. When the load was up to 420 kN, a long main fracture appeared near the mid-span section of the test beam. When the load reached 490 kN, the main fracture crossed through the section of concrete slab and the crack width reached 0.1 mm. With the increase of load, there were more and more cracks near the mid-span section of the test beam. At last, eight main concrete cracks occurred, as shown in Figure 8a, which were distributed near the intermediate supported section of test beam. The average length of the concrete cracks was about 1100 mm, and the maximum width of the concrete cracks was 0.2 mm.

(2) Specimen SPB

At the beginning stage of loading, the specimen SPB behaves well in an elastic state. When the load reached 620 kN, some short concrete cracks occurred near the mid-span section of test beam. When the loading value reached 800 kN, the previous short crack developed slightly. With the increase of load, a number of short cracks appeared again in the concrete slab. The maximum value of crack width was up to 0.02 mm, when the loading up to 900 to kN. When the load reached 980 kN, the density of short cracks increased, distributed within a wide length range of 1800 mm, and the maximum value of crack width for most concrete cracks was 0.02 mm. As shown in Figure 8b, no obvious main cracks appeared, and no cracks crossed through the concrete slab during the loading process.

(3) Comparison of crack property on two experimental beams

The crack property of the concrete slab of the experimental beams is shown in Table 4. The cracking morphology of the two experimental beams after test completion is shown in Figure 8.

In light of Table 4 and Figure 8, which describe the concrete slab cracking state of test beam, the following analysis conclusions can be drawn.

1. The initial cracking load of specimen SPB was 107% higher than that of specimen SRB, indicating that the interface slip effect between steel and concrete of specimen SPB was more pronounced, due to the use of URSP connectors, which improves crack resistance.
2. SPB specimens had a slower fracture follow-up rate than SRB specimens. When the loading value of specimen SRB reached 490 kN, the first main fracture ran through the concrete slab, and a total of 8 main fractures were generated, with a maximum fracture width of 0.2 mm. However, as a result of loading, specimen SPB did not show any obvious main cracks and had a maximum crack width of 0.02 mm. This indicates that the interface slip effect can slow down the crack development speed of concrete slab and effectively reduce the crack width under the same loading conditions.
3. In terms of fracture distribution, specimen SPB had a wider fracture distribution range. It indicates that the tensile stress distribution of specimen SPB is more uniform than that of specimen SRB.

## 4. Comparative Analysis of Stiffness, Interfacial Slip, Prestress Efficiency, and Other Test Indicators of Two Experimental Specimens

(1) Comparative analysis of stiffness

The mid-span section with the most obvious deflection variation was selected as the observation point to analyze the influence of structural stiffness on structural deformation, so as to reflect the stiffness differences between the two types of experimental beams.

**Figure 9:**
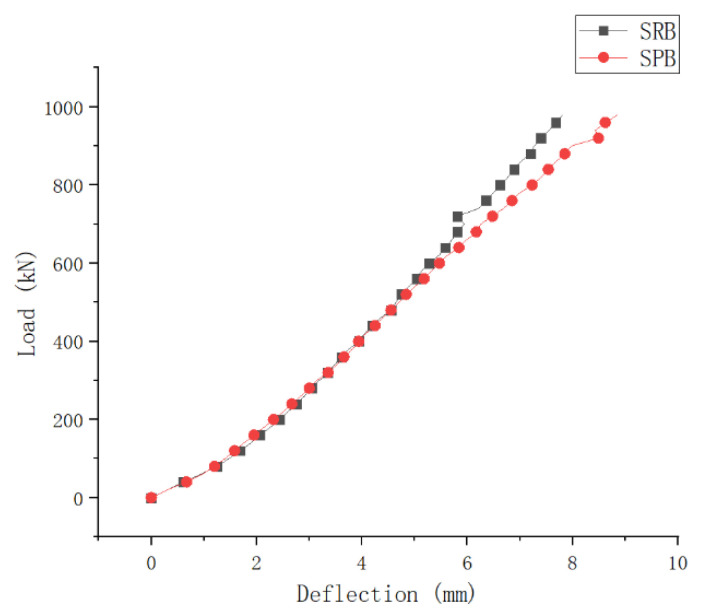
Load-Deformation Curve of Experimental Beam.

Figure 9 shows load-deformation curves for specimens SRB and SPB.

According to Figure 9, specimens SRB and SPB showed similar deflection changes during early loading. However, specimen SPB exhibited higher deflection at the late loading stage than specimen SRB. At maximum loading, specimen SPB had a greater load deformation at the intermediate support section than specimen SRB by 13.4%. It indicates that the upper concrete slab and the lower steel structure did not form a combined structure, due to the interface slip that occurred in the negative moment zone. Because both of test beams deformed in a similar manner during early loading stage, it shows that both of them had the same overall stiffness during the early loading stage. During the late loading stage, the interface slip had a significant effect on the overall structure stiffness, and the structural deformation of specimen SPB was slightly larger than that of specimen SRB, but the difference was not obvious.

(2) Comparative analysis of interface slip

**Figure 10:**
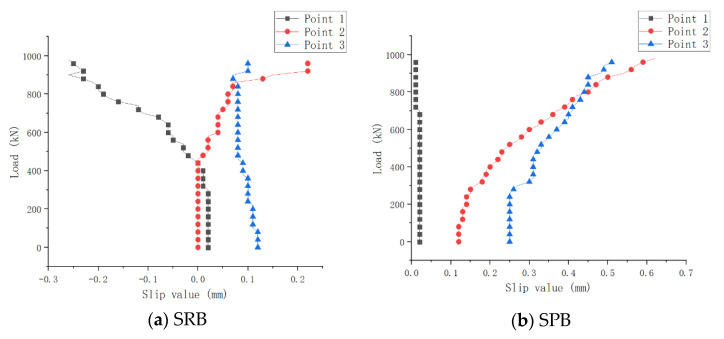
Variation curves of load-interface slip deformation.

The setting of three measuring points of interface slip in the negative moment zone is shown in Figure 6. The variation of slip value at each measuring point in the loading stage are shown in Figure 10. It is assumed that the relative displacement between the concrete slab and steel portion in the figure is positive to the left.

After prestressing, each measuring point’s slip value was set as the initial slip value. Figure 10 shows that a closer distance between each measuring point and both ends of the specimen means a greater initial slip value.

The slip value of specimen SRB with the traditional connector was small, and with the loading increases, the relative slip of the measuring point 1 also occurred in the opposite direction. It shows that the direction of the relative displacement between the concrete and steel turned to the right, instead of to the left, when the concrete cracked at the mid-span section.

The uplift-restricted and slip-permitted connectors were used in specimen SPB, and the initial slip value and subsequent slip value were larger, which achieved the design purpose of “uplift-restricted and slip-permitted connecting” by new shear connectors. The slip value of measuring point 1 at the mid-span section was always small, and the initial slip value of measuring point 2 was less than that of measuring point 3. However, the slip value of measuring point 2 increased faster during the loading process. After the loading value reached 780 kN, the slip value of measuring point 2 slightly exceeded that of measuring point 3. Because both ends of the experimental beam were constrained by two roller supports, a large normal stress was generated between the steel–concrete interface, which increased the sliding friction at both ends of the experimental beam. Therefore, in the late loading stage, the interface slip value of measuring point 3 was slightly lower than that of measuring point 2, due to the effect of interface friction. However, in general, except for the mid-span section, the mechanical effect of interfacial slip in other regions was still obvious.

(3) Comparative analysis of prestress efficiency

**Figure 11:**
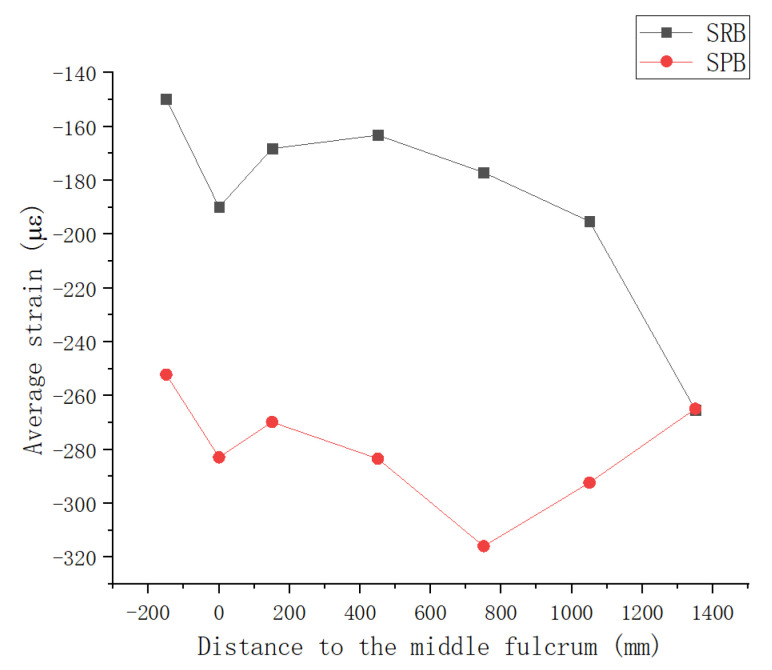
Longitudinal Distribution of Average Strain on Top of Concrete Deck at 7 Cross-Sections.

**Figure 12:**
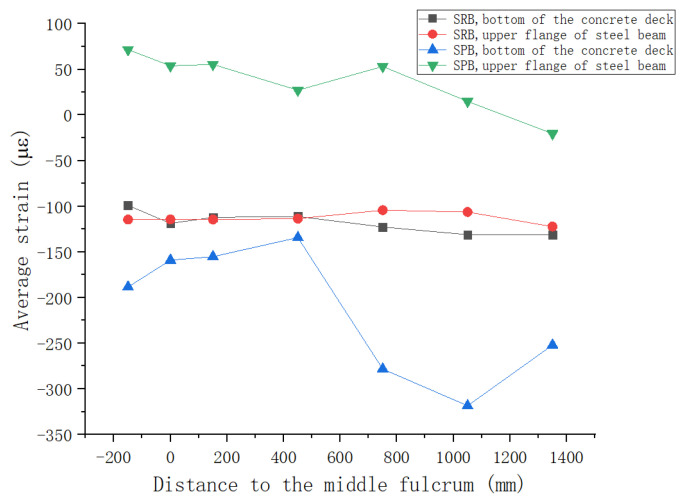
Longitudinal Distribution of Fiber Strain Values Adjacent to the Interface of Steel–concrete for 7 Cross-Sections.

The experimental beam was configured with strain gauges in seven cross-sections. The specific arrangements of strain gauges are shown in Figure 5. In Figure 11, the longitudinal distribution of the strain mean on the top surface of the concrete slab after prestressing is shown, while the longitudinal distribution of fiber strain values for seven cross-sections, which were adjacent to the interface of steel and concrete along the longitudinal direction, are shown in Figure 12.

As can be seen from Figure 11, except for the section near the end of experimental beam, the fiber compressive strain on the top surface of the concrete slab of specimen SPB was higher than that of specimen SRB. This indicates that the interface slip causes the specimen SPB to store greater precompression stress in the top fiber of the concrete slab after prestressing, that is, specimen SPB had a higher prestressing efficiency than specimen SRB.

Figure 12 shows the strain distribution of the concrete and steel portion near the steel–concrete interface of composite girder. If the strain values of the two portions differed greatly, it indicates that the relative slip between the two portions near the interface was more obvious.

As can be seen from Figure 12, for SRB specimens with traditional connectors, the compressive strains of the two portions near the interface were basically the same, with little difference between them, indicating that the traditional connectors make concrete and steel to form a complete composite section. Based on the strong connection between them, the external pre-pressure applied to the concrete slab was distributed evenly from the upper concrete slab to the lower steel portion through the traditional connectors, but it was inconsistent with the design objective of the prestressing applied. This phenomenon reduces the prestressing efficiency of steel–concrete composite girder with traditional connectors.

According to the strain monitoring data corresponding to the SPB beam in Figure 12, the longitudinal distribution of fiber strains adjacent to the steel–concrete interface of SPB beams differed significantly from that of the SRB beams, due to the adoption of a new shear connector, and the bottom surface of concrete slab had a higher compressive strain than its lower steel portion.

The results indicate that the SPB beam had significant interface slip, resulting in almost no prestress transferring to the upper flange of the steel beam, so that most prestress was borne by the concrete slab. The prestress efficiency of the specimen SPB was significantly improved, and the crack resistance of the concrete slab of the composite girder was also improved. The mechanical properties mentioned above are consistent with the design objective of applying prestress, and it is evident that increasing the interface slip improves the crack resistance of concrete slab within the negative moment region of continuous composite beams.

(4) Comparative analysis of longitudinal strain distribution at the mid-span section

**Figure 13:**
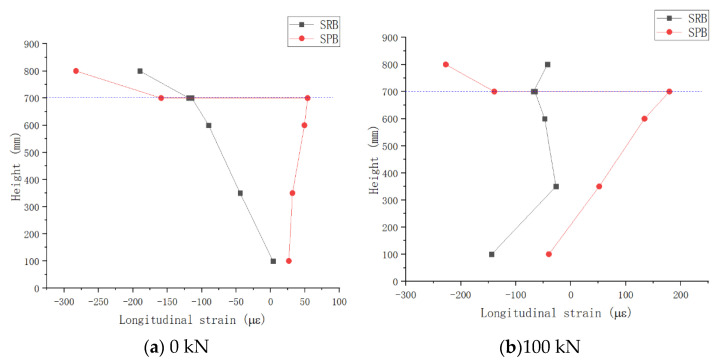

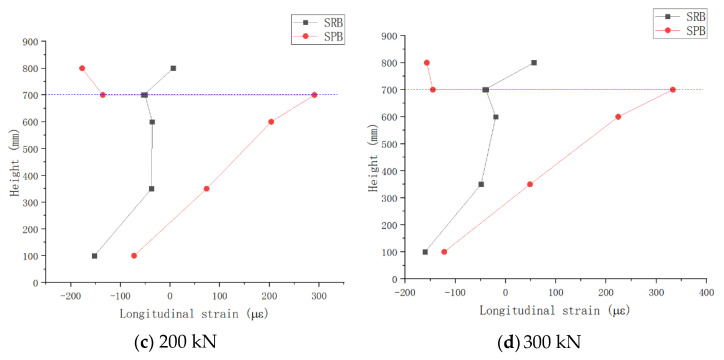
Vertical Distribution of Longitudinal Strain of Steel Box Girder Web.

The layout of strain gauge at the mid-span section is shown in Figure 5. Four loading steps, with external loads of 0 kN, 100 kN, 200 kN, and 300 kN, were selected for comparative analysis. The strain distribution at the mid-span section is shown in Figure 13. Strain was positive in tension and negative in pressure. As shown in the figure, the ordinate height represents the distance between the measuring point and the bottom of the steel beam. The height of 700 mm in the figure is the dividing line between the concrete slab and steel portion of the composite girder. The data above the dividing line are the longitudinal strains along the concrete slab, and below the line are the longitudinal strains along the steel web.

According to the data analysis in Figure 13, it can be seen that:

1. In each loading stage, the longitudinal strains of the bottom surface of the concrete slab and the upper flange plate of the steel portion of specimen SRB were basically the same, indicating that the concrete slab and the steel portion formed a combined section to work together. However, the longitudinal strain of concrete slab and steel portion of SPB was much different from that of SRB, and both had specific strain distribution characteristics, respectively. This shows that two portions in the negative bending moment region of specimen SPB formed an ideal laminated beam, and the steel structure and concrete slab bore the load, respectively.
2. Figure 13a shows that specimen SRB concrete slab had a smaller compressive strain under prestressing alone than specimen SPB, while the compressive strain on the top surface of steel portion of specimen SRB was significantly larger than that of specimen SPB, indicating that specimen SRB exhibited a significantly greater compressive strain on the top surface of steel than specimen SPB. However, SPB showed large longitudinal strain gaps between the concrete slab and steel portion, and there were no compressive strains in most sections of the steel portion. Through the new type of connectors, the interface slip effect prevented the prestress exerted on the concrete slab from transferring to the steel portion, and the concrete slab itself bore most of the prestress, thus improving the prestress efficiency of composite girder.
3. In the subsequent loading stages, the compressive strain of the concrete slab in specimen SRB was smaller than that of concrete slab in specimen SPB, and the tensile strain of the steel portion in specimen SRB was smaller than that in the specimen SPB steel portion. The above facts show that most of the negative bending moment is borne by the steel beam due to the interface slip effect, thus reducing the tensile stress borne by the concrete slab.
4. It is obvious that the increase in tensile strain on the top surface of concrete slabs in specimen SPB was smaller than that in specimen SRB as applied load increases, so the interface slip effect caused specimen SPB have a better crack resistance.

(5) Analysis on strain on top surface of concrete slab at mid-span section

**Figure 14:**
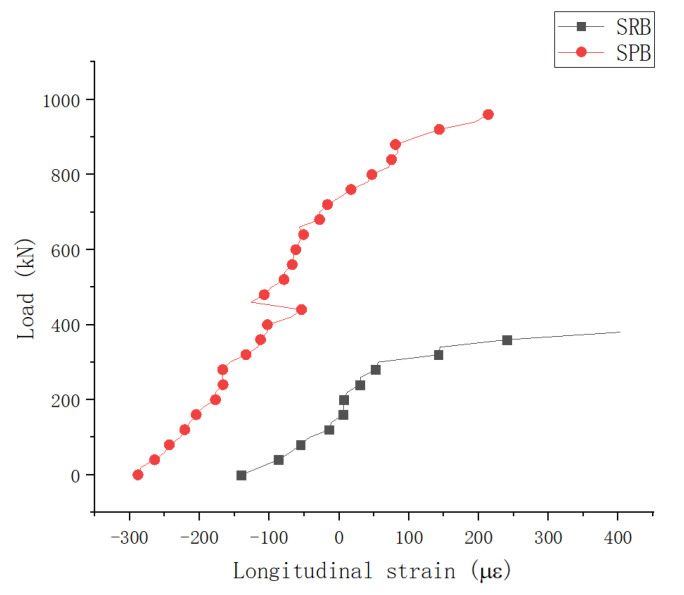
Load-Strain Curve of the Concrete Top Surface at the Middle Fulcrum Section.

Figure 14 shows the average strain variation in the mid-span section of the concrete slab.

As can be seen from Figure 14, since the interface slip effect improved the prestressing efficiency of specimen SPB, before external loading, the compressive strain of specimen SPB was greater than that of specimen SRB (i.e., when the external load was 0 kN). During loading, specimen SPB showed significantly lower tensile strain than specimen SRB, and the increasing curvature of the tensile strain was low. Hence, the interface sliding effect not only improves prestressing efficiency in continuous composite girders’ negative moment zones, but the tensile strain in the specific region is also reduced during subsequent loading. As a result of this mechanical effect, the specimen SPB cracked later and developed cracks slower in the negative bending moment region.

(6) Comparative analysis of the longitudinal distribution of strain on the top surface of concrete slab

**Figure 15:**
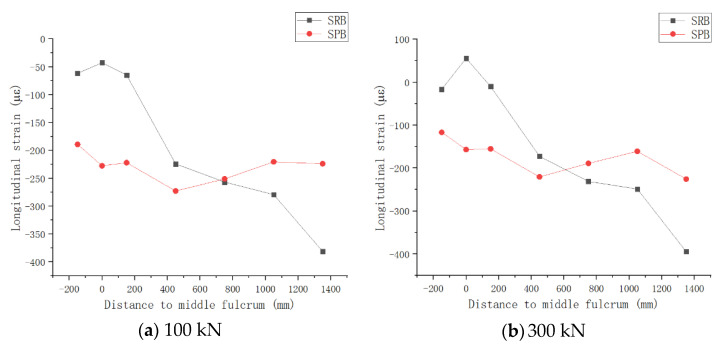
Longitudinal Distribution of Average Strain of Concrete Deck under Negative Bending Moment.

A concrete slab with seven longitudinal sections is taken as the object in Figure 5a. The longitudinal distributions of the strains at each measuring point of two specimens were compared and analyzed with each other, in order to determine the variation law of the tensile stress of the concrete slab in the subsequent loading process and the influence mechanisms of the interface slip effect on it in the negative moment zone of continuous composite beams. Figure 15 shows the average strain distribution for two specimens before cracking under two load steps of 100 kN and 300 kN.

Figure 15 shows that the stress strain on the top surface of the concrete slab of two specimens was a compressive strain, as the prestress was applied before the external load applied, and the structure presented a state of compression. However, with the subsequent action of the external load, the compressive strain in the concrete slab gradually decreased, and the tensile strain also gradually appeared, which lead to the gradual cracking of some sections. Based on the analysis of strain distribution, it can be seen that there were significant differences in the mechanical characteristics between the two specimens. First of all, under the same grade of load, the interface slip made the strain distribution of specimen SPB in the whole negative moment region more uniform. Even if the specimen SPB cracked under the action of subsequent tensile stress, the crack distribution was more uniform, the distribution range was wider, and the crack width was smaller. Secondly, for the specimen SRB with traditional shear connectors, the tensile stress of the concrete slab was mainly concentrated near the mid-span section, which made it easy to cause concrete cracking. For specimen SPB, the interface slip prevented the concentration of tensile stress near the mid-span section and reduced the peak value of tensile stress. As a result, the tensile stress in the slab’s negative moment zone was distributed uniformly, which, in fact, reduced the cracking possibility of specimen SPB. Even if specimen SPB cracked, the crack width grew more slowly after cracking, which improved the durability of the continuous composite structure in the negative moment region.

## 5. Conclusions

In order to study the feasibility and the working mechanism of improving the crack resistance of continuous steel–concrete composite bridge by releasing the interfacial slip effect within the negative bending moment region, two group of model tests were carried out in the paper. The main conclusions are as follows.

(1) The model tests show that the crack resistance of continuous composite beams in the negative moment region can be improved significantly by releasing the interface slip. The cracking load of composite structure rises by 107% when the continuous composite structure uses the uplift-restricted and slip-permitted connectors in the negative moment zone, due to releasing its interface slip. Moreover, the interface slip can make the tensile stress distribution more uniform on the concrete slab within the negative bending moment region and avoid the concentration of tensile stress in the mediate support, resulting in a wider distribution of cracks in the bridge deck within the negative bending moment region and a slower development rate of cracks.
(2) Compared with the composite beam with conventional shear studs, the composite beam with Uplift-Restricted and Slip-Permitted shear connectors has a higher pre-stress application efficiency, due to an important fact the new connector could release the interface slip. As the interface slip is released, the applied prestress can be effectively retained in the concrete slab, preventing the prestress from being transferred to the steel structure through the shear connectors.
(3) Another working mechanism in which the releasing interface slip improves the crack resistance of the structure is that, during the loading stage, specimen SPB behaves with lower values of both tensile strain and tensile strain growth curvature than specimen SRB, and the tensile strain distribution in the concrete slab of specimen SPB is more uniform along the longitudinal direction. This indicates that releasing the interface slip can effectively slow down the increase of the tensile strain of the concrete slab during the loading process after cracking.
(4) Despite the application of uplift-restricted and slip-permitted connectors in the hogging moment zone, the structural stiffness of the SPB beam was not significantly different from that of the continuous composite structure with traditional stud connectors, indicating that, in the negative moment zone of the continuous composite box girder, the releasing interface slip had no significant effect on structural stiffness.

## Figures and Tables

**Table 1:** Test strength of concrete (unit: MPa).

Number	Curing Days/d	Cubic Strength	Cleavage Strength	Young’s Modulus
SRB	28	58.25	3.30	35,778
SPB	28	58.7	3.35	35,805

**Table 2:** Test strength of steel plate.

Thickness/mm	Element	Yield Strength/MPa	Ultimate Strength/MPa
6	Flange, web	349.55	488.05
4	Stiffening rib	365.36	538.92

**Table 3:** Test strength of reinforcements (strength unit: MPa).

Diameter/mm	Component	Yield Strength	Ultimate Strength
6	Transverse reinforcement	349.55	488.05
8	Longitudinal reinforcement	365.36	538.92
15.24	Prestressed reinforcement	1780.00	1923.00

**Table 4:** Cracking of experimental beam.

Specimen	Load (kN)	Phenomena
SRB	300	First crack occurred on concrete slab at the mid-span section.
	380	A large number of cracks occurred at the left and right sides of the concrete slab.
	420	A long main crack occurred on concrete slab at the mid-span section.
	440	A main crack spread across the entire concrete slab at the midspan section.
	490	In the middle span section, the main crack penetrated the whole slab, reaching a maximum width of 0.1 mm.
	600	On both sides of the main crack in the mid-span section, two new main cracks appeared. The maximum width of the main crack was 0.2 mm.
	640	The maximum width of two new cracks reached 0.1 mm, the others continued.
	780	In the area near the midspan section of the concrete slab, 6 new cracks developed and old cracks expanded.
	900	There was a maximum width of 0.2 mm for one main crack at mid-span section, and 0.1 mm for four main cracks on both sides of the main crack.
SPB	620	Two short cracks occurred on the right top surface of the concrete slab at the mid-span section.
	760	At the midspan section of the concrete slab, three short cracks appeared.
	800	A short crack had developed slightly and was 0.02 mm wide at its widest point.
	900	Concrete slab top surface developed many short cracks with short lengths, averaging 0.02 mm in width.
	980	The density of short cracks increased, there was no obvious main crack, and there were some cracks occurred in a zone near the side support.

## Data Availability

All the data is available within the manuscript.

## References

[1] Su Q.-T., Yang G.-T., Wu C. (2012). Experimental investigation on inelastic behavior of composite box girder under negative moment. Int. J. Steel Struct..

[2] De Maio U., Greco F., Leonetti L., Blasi P.N., Pranno A. (2022). A cohesive fracture model for predicting crack spacing and crack width in reinforced concrete structures. Eng. Fail. Anal..

[3] Fan J., Gou S., Ding R., Zhang J., Shi Z. (2020). Experimental and analytical research on the flexural behaviour of steel–ECC composite beams under negative bending moments. Eng. Struct..

[4] Qi J., Cheng Z., Wang J., Tang Y. (2020). Flexural behavior of steel-UHPFRC composite beams under negative moment. Structures.

[5] Zhang Y., Cai S., Zhu Y., Fan L., Shao X. (2020). Flexural responses of steel-UHPC composite beams under hogging moment. Eng. Struct..

[6] Fan J., Nie X., Li Q., Li Q. (2010). Long-Term Behavior of Composite Beams under Positive and Negative Bending. II: Analytical Study. J. Struct. Eng..

[7] Fan J., Nie J., Li Q., Wang H. (2010). Long-Term Behavior of Composite Beams under Positive and Negative Bending. I: Experimental Study. J. Struct. Eng..

[8] El-Zohairy A., Salim H., Saucier A. (2019). Steel–Concrete Composite Beams Strengthened with Externally Post-Tensioned Tendons under Fatigue. J. Bridg. Eng..

[9] Nie J.-G., Li Y.-X., Tao M.-X., Nie X. (2015). Uplift-Restricted and Slip-Permitted T-Shape Connectors. J. Bridg. Eng..

[10] Nie J., Wang J., Gou S., Zhu Y., Fan J. (2019). Technological development and engineering applications of novel steel-concrete composite structures. Front. Struct. Civ. Eng..

[11] He J., Liu Y., Chen A., Yoda T. (2010). Experimental study on inelastic mechanical behaviour of composite girders under hogging moment. J. Constr. Steel Res..

[12] Saari W.K., Hajjar J.F., Schultz A.E., Shield C.K. (2004). Behavior of shear studs in steel frames with reinforced concrete infill walls. J. Constr. Steel Res..

[13] Shim C.-S., Lee P.-G., Yoon T.-Y. (2004). Static behavior of large stud shear connectors. Eng. Struct..

[14] Lee P.-G., Shim C.-S., Chang S.-P. (2005). Static and fatigue behavior of large stud shear connectors for steel–concrete composite bridges. J. Constr. Steel Res..

[15] Tan Y., Zhu B., Yan T., Huang B., Wang X., Yang W., Huang B. (2019). Experimental Study of the Mechanical Behavior of the Steel–Concrete Joints in a Composite Truss Bridge. Appl. Sci..

[16] Nie J.G., Tao M.X. (2012). Slab spatial composite effect in composite frame systems. I: Effective width for ultimate loading capacity. Eng. Struct..

[17] Bursi O.S., Sun F.-F., Postal S. (2005). Non-linear analysis of steel–concrete composite frames with full and partial shear connection subjected to seismic loads. J. Constr. Steel Res..

[18] Wang J., Wang W., Lehman D., Roeder C. (2019). Effects of different steel-concrete composite slabs on rigid steel beam-column connection under a column removal scenario. J. Constr. Steel Res..

[19] Amadio C., Bedon C., Fasan M. (2017). Numerical assessment of slab-interaction effects on the behaviour of steel-concrete composite joints. J. Constr. Steel Res..

[20] Campi F., Monetto I. (2013). Analytical solutions of two-layer beams with interlayer slip and bi-linear interface law. Int. J. Solids Struct..

[21] Zhuang B., Liu Y., Yang F. (2018). Experimental and numerical study on deformation performance of Rubber-Sleeved Stud connector under cyclic load. Constr. Build. Mater..

[22] Wang S., Tong G., Zhang L. (2017). Reduced stiffness of composite beams considering slip and shear deformation of steel. J. Constr. Steel Res..

[23] Kelkel B., Popow V., Gurka M. (2010). FE model to simulate bond-slip behavior in composite concrete beam bridges. Comput. Struct..

[24] Xu X., Liu Y. (2016). Analytical and numerical study of the shear stiffness of rubber-sleeved stud. J. Constr. Steel Res..

[25] Abe H., Hosaka T., Hajjar J.F., Hosain M., Easterling W.S., Shahrooz B.M. Flexible Shear Connectors for Railway Composite Girder Bridges. Proceedings of the Composite Construction in Steel and Concrete IV Conference 2000.

[26] Ding Y., Dai X.M., Yan J.B. (2019). Developments and behaviors of slip-released novel connectors in steel-concrete composite structures. Adv. Steel Constr..

[27] Chen Y., Yan Q., Yu X., Jia B., Wu Y., Luo Y. (2022). Experimental and Numerical Research on Uplift-Restricted and Slip-Permitted Screw-Shaped Connectors. Int. J. Steel Struct..

[28] Duan L., Nie X., Ding R., Zhuang L. (2019). Research on Application of Uplift-Restricted Slip-Permitted (URSP) Connectors in Steel-Concrete Composite Frames. Appl. Sci..

[29] Li Z.-Y., Tao M.-X., Nie J.-G., Fan J.S. (2017). Analysis and optimization of a continuous composite bridge with uplift-restricted and slip-permitted connectors. IABSE Symposium Report.

[30] Duan L.L., Chen H.B., Nie X., Han S.W. (2020). Experimental study on steel-concrete composite beams with Uplift-restricted and slip-permitted screw-type (URSP-S) connectors. Steel Compos. Struct..

